# Upper Gastrointestinal Bleed due to Duodenal Metastases of Lung Adenocarcinoma: Report of Two Cases and Review of Literature

**DOI:** 10.1155/2019/3437056

**Published:** 2019-10-20

**Authors:** Sumitro Kosasih, Khairul Najmi Muhammad Nawawi, Zhiqin Wong, Deborah Chew Chia Hsin, Andrea Yu-Lin Ban, Raja Affendi Raja Ali

**Affiliations:** ^1^Gastroenterology Unit, Department of Medicine, Faculty of Medicine, The National University of Malaysia Medical Centre, Kuala Lumpur, Malaysia; ^2^Respiratory Unit, Department of Medicine, Faculty of Medicine, The National University of Malaysia Medical Centre, Kuala Lumpur, Malaysia

## Abstract

Upper gastrointestinal bleeding as a result of gastrointestinal metastases from lung cancer is extremely rare. We report two cases of patients with duodenal metastases from lung adenocarcinoma presented with recurrent melena. Histopathological examination and immunohistochemical staining of the duodenal biopsies supported the diagnosis of metastatic lung adenocarcinoma.

## 1. Introduction

Upper gastrointestinal bleeding (UGIB) is a medical emergency and usually treated via either endoscopic, radiological, or surgical intervention. We present two cases of a rare cause of UGIB secondary to duodenal metastases of lung cancer. In lung cancer, distant metastases are more commonly found in the adrenal glands, bone, liver, and brain. However, metastasis in the small bowel is extremely uncommon, in which jejunum is the most frequent site of involvement (50.9%), followed by ileum (33.3%) and duodenum (15.8%) [1]. Initial presentation with UGIB of lung cancer due to duodenal metastasis is exceedingly uncommon.

## 2. Case Report 1

**Figure 1:**
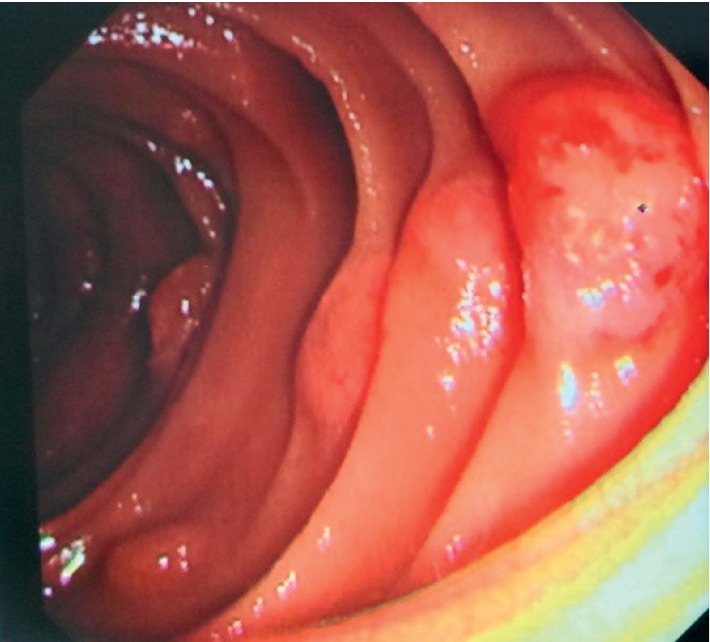
OGDS showed multiple polypoidal lesions with central ulceration at the second part of duodenum.

**Figure 2:**
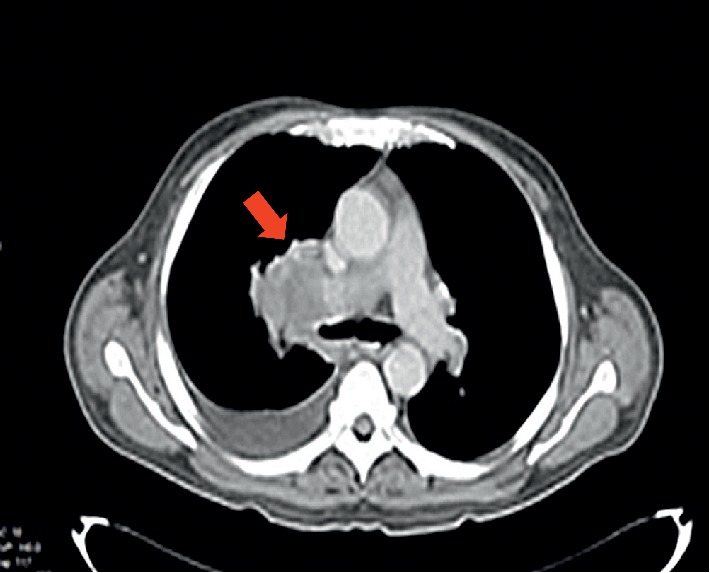
CT thorax showed a right upper intrabronchial neoplastic lesion (red arrow) with right pleural effusion.

**Figure 3:**
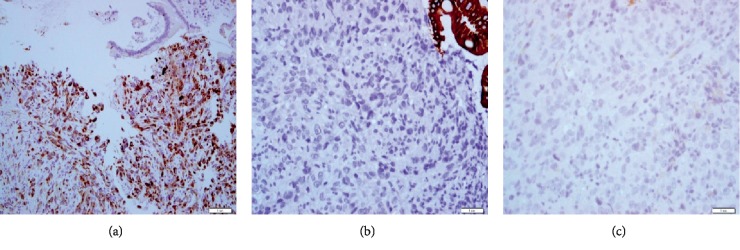
Immunohistostaining of the duodenal biopsies demonstrated (a) CK7-positive, (b) CK20-negative, and (c) TTF1-positive.

A 64-year-old man, active smoker, presented with melena, weakness, and exertional dyspnea. He also complained of hemoptysis and weight loss for the past two months. His only medication was nonsteroidal anti-inflammatory drugs. On physical examination, he was noted to be pale. He was tachycardic but had normal blood pressure. Chest examination revealed right lower zone crepitations and multiple cervical and axillary lymphadenopathies. The initial laboratory examinations showed normochromic normocytic anemia with hemoglobin of 4.1 g/dL and raised white cell count of 23 × 10^9^/L, sodium 120 mmol/L, potassium 4.5 mmol/L, urea 12.3 mmol/L, creatinine 143.5 *μ*mol/L, and C-reactive protein at 10.76 mg/dL. Based on the finding of his chest X-ray, he was treated for community-acquired pneumonia. In consideration of melena and anemia, he underwent an oesophago-gastroduodenoscopy (OGDS), which showed multiple polypoidal lesions with central ulceration at the second part of duodenum (Figure 1). A computed tomography (CT) scan of the thorax revealed a right upper lobe bronchogenic tumor with liver, mesenteric, and contralateral lung metastases (Figure 2). Histopathological examination of the duodenum revealed lung adenocarcinoma in which immunohistostaining was positive for both cytokeratin 7 (CK7) and thyroid transcription factor 1 (TTF1), but negative for CK20 (Figure 3). He also underwent a fine needle aspiration of the cervical lymph nodes, in which it revealed lung adenocarcinoma.

While inpatient, his condition deteriorated with ongoing sepsis and persistent episodes of melena, which was not manageable by both endoscopy and radiological interventions. Hence, he was managed conservatively with the involvement of palliative care team.

## 3. Case Report 2

A 73-year-old lady, nonsmoker, presented with a week history of generalized weakness, exertional dyspnea and multiple episodes of melena. The patient had a medical history of underlying hypertension and diabetes mellitus. She had two previous admissions for melena and pneumonia in the past couple of months, necessitating blood transfusions and intravenous antibiotics. Initial laboratory investigations showed hypochromic microcytic anemia with hemoglobin of 7.0 g/dL, platelet 634 × 10^9^/L, albumin 23 g/L, and normal white cell counts. Physical examination revealed signs of anemia and reduced breath sound on the left lower zone of the lungs with no lymphadenopathy or organomegaly.

**Figure 4:**
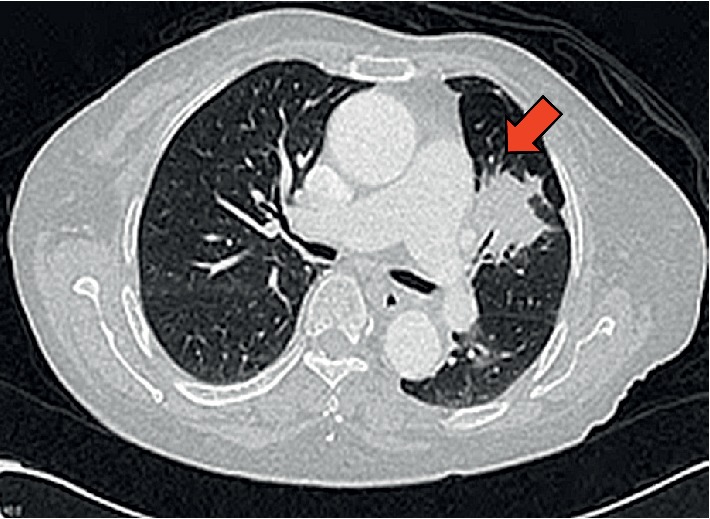
CT thorax showed a left upper lobe neoplastic lesion (red arrow) with multiple lymphadenopathies in the mediastinum.

**Figure 5:**
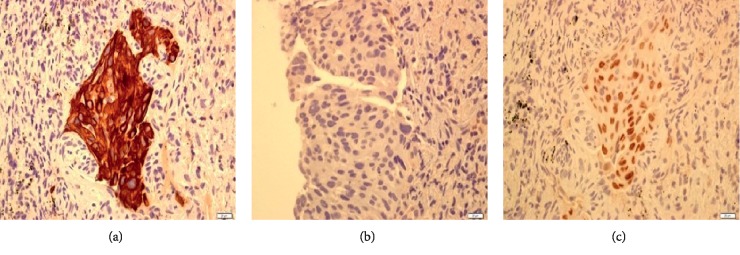
Immunohistostaining of neoplastic lesion in the lung demonstrated a positive for CK-7 (a), negative for CK-20 (b), and positive for TTF-1 (c).

An OGDS revealed a large circumferential mass measuring 2.5 cm with ulcerated base at the distal duodenum. Histopathological examination confirmed a duodenal metastasis with poorly differentiated adenocarcinoma. Immunohistostaining was positive for CK7 but negative for TTF1 expression. CT thorax was performed (Figure 4) and showed left upper lobe lung mass measuring 3.9 × 3.2 × 4.0 cm with multiple mediastinal and abdominal lymphadenopathies as well as evidence of liver metastases. Histopathological examination of her lung mass (CT guided biopsy) was consistent with lung adenocarcinoma which was CK7 and TTF1-positive and CK20-negative (Figure 5). On further follow-up on the patients, she had no further melena and was planned for chemo-radiotherapy for the metastatic lung adenocarcinoma.

## 4. Discussion

Lung cancer is the second leading cause of malignancy among Malaysian males with the incidence rising to 15.8% of all cancer [2]. It is also the third most common overall malignancy (combined males and females) diagnosed in Malaysia according to the Malaysian National Cancer Registry Report (2007–2011) after breast and colorectal carcinoma [2]. Lung cancer has various clinical presentations such as nonspecific respiratory symptoms, paraneoplastic syndromes, and/or symptoms occurring as the result of local or distant metastases.

Lung cancer with gastrointestinal tract metastasis occurs at 0.3–1.8% [3–6]. However, a much higher incidence has been noted at autopsies. In a large case series involving 11-year period of assessment, McNeill et al. found 46 out of 431 patients (11%) with primary lung cancer who underwent autopsy had small bowel metastases. During the same period, they examined 78 surgical specimens from the small bowel with metastatic tumor and noted that 6 specimens (7.6%) were from lung primaries [7]. In a more recent review by Yoshimoto et al., they identified 11.9% cases with gastrointestinal metastasis [8]. Multiple metastases occurred in 6.2% in the stomach, 5.1% in the small bowel, and 4.5% in the large bowel. Among the small bowel metastases, duodenal involvement is the least common at 0.9%.

In another large case series involving patients with non-small cell lung cancer, 10 out of 218 autopsies were found to have small bowel metastases (incidence of 4.6%). Notably, all of them already had concurrent other metastatic sites, which include adrenal (90%), mediastinal lymph nodes (80%), liver (70%), pleura (60%), contralateral lung (60%), and bones (60%) [9]. This indicates the advanced stage of the primary lung cancer, once the small bowel metastases are detected.

Primary lung cancer with small bowel metastases is mostly asymptomatic. However, in small reported cases, these metastases can cause perforation of the small bowel due to tumor invasion to the bowel wall, obstruction, or intussusception of small bowel because of rapidly growing metastatic tumor [3, 4, 10–14]. Bowel perforation carries a dire prognosis as shown in a case series of 15 patients with bowel perforation, in which no patient has survived more than 16 weeks following operation [7]. In our 2 cases, both patients presented with melena and duodenal metastasis of lung cancer, which also had a dismal prognosis, with an average survival of 2.6 months.

UGIB due to duodenal metastasis of lung cancer is uncommon. Hence, standard treatment has yet to be formulated. Several treatment modalities reported include surgery, endoscopic resection, and chemo-radiotherapy. Hirai et al. reported successful surgical resection for metachronous metastases in duodenum and jejunum with survival up to 6 years on follow-up. However, Lee et al. reported surgical mortality rate of 22% for patients with gastrointestinal metastasis of lung cancer [15–18]. Endoscopic resection [19, 20] has been reported in several cases with metastatic tumor of size less than 1 cm. Ito et al. had demonstrated that chemo-radiotherapy regimen with four cycles of cisplatin and etoposide followed by abdominal irradiation at a dose of 30 Gy to patients with small cell lung cancer with duodenal metastasis showed reasonably good partial response [21]. Zhou et al. highlighted the potential benefit of tyrosine kinase inhibitors (TKI) in primary lung cancer with driver gene mutations in gastrointestinal metastases. In their case series of three patients, all patients survived for another on average 12 months following introduction of TKIs (crizotinib, icotinib, and brigatinib) after detection of the relapse. They also emphasized the need of re-biopsy after the initial chemotherapy and performing large panel of gene mutation detection such as epidermal growth factor receptor (EGFR), ROS1, and mesenchymal-to-epithelial transition (MET). In regard to the diagnosis, Rossi et al. recommended the use of immunohistostaining of cytokeratins 7 and 20 (CK7 and CK20), thyroid transcription factor 1 (TTF1), and caudal-related homeobox 2 (CDX2) in the gastrointestinal histologic samples. This application is helpful in highlighting the lung primary [22].

In our first case, the patient had recurrent UGIB despite the combination of endoscopic and radiological interventions. He was, however, unfit for any surgical intervention. He succumbed to his disease due to severe pneumonia and ongoing bleeding. In our second case, the UGIB stopped spontaneously without any intervention, and endoscopic resection was not possible due to the large size of the tumor. Hence, the patient was planned for concurrent chemo-radiotherapy (chemotherapy regimen: cisplatin-paclitaxel and radiotherapy at a dose of 30 Gy). Since UGIB had ceased, surgical intervention was not offered to the patient, as the previously reported cases had shown a high mortality rate of up to 22% [15, 17].

## 5. Conclusion

Duodenal metastasis of lung cancer as the cause of UGIB is extremely uncommon but has to be considered and always taken into account, as one of the differential diagnosis. Treatment of duodenal metastasis is yet to be established, and it remains challenging depending on the site and size of the metastatic tumor. The primary aim is to treat the underlying disease (lung cancer in our case) and to achieve hemostasis by either endoscopic, radiological, or surgical intervention.
